# Bioactive magnetic near Infra-Red fluorescent core-shell iron oxide/human serum albumin nanoparticles for controlled release of growth factors for augmentation of human mesenchymal stem cell growth and differentiation

**DOI:** 10.1186/s12951-015-0090-8

**Published:** 2015-05-07

**Authors:** Itay Levy, Ifat Sher, Enav Corem-Salkmon, Ofra Ziv-Polat, Amilia Meir, Avraham J Treves, Arnon Nagler, Ofra Kalter-Leibovici, Shlomo Margel, Ygal Rotenstreich

**Affiliations:** Department of Chemistry, Bar-Ilan Institute of Nanotechnology and Advanced Materials, Ramat-Gan, 52900 Israel; Goldschleger Eye Institute, Sackler Faculty of Medicine, Tel Aviv University, Sheba Medical Center, Tel-Hashomer, 52621 Israel; Center for Stem Cells and Regenerative Medicine, Cancer Research Center, Sheba Medical Center, Tel-Hashomer, 52621 Israel; Hematology Division, Sheba Medical Center, Tel-Hashomer, 52621 Israel; Unit of Cardiovascular Epidemiology, Gertner Institute for Epidemiology and Health Policy Research, Ramat Gan, Israel, Sackler Faculty of Medicine, Tel-Aviv University, Tel-Aviv, Israel

**Keywords:** IO/HSA NPs, FGF2, BM-MSCs

## Abstract

**Background:**

Iron oxide (IO) nanoparticles (NPs) of sizes less than 50 nm are considered to be non-toxic, biodegradable and superparamagnetic. We have previously described the generation of IO NPs coated with Human Serum Albumin (HSA). HSA coating onto the IO NPs enables conjugation of the IO/HSA NPs to various biomolecules including proteins. Here we describe the preparation and characterization of narrow size distribution core-shell NIR fluorescent IO/HSA magnetic NPs conjugated covalently to Fibroblast Growth Factor 2 (FGF2) for biomedical applications. We examined the biological activity of the conjugated FGF2 on human bone marrow mesenchymal stem cells (hBM-MSCs). These multipotent cells can differentiate into bone, cartilage, hepatic, endothelial and neuronal cells and are being studied in clinical trials for treatment of various diseases. FGF2 enhances the proliferation of hBM-MSCs and promotes their differentiation toward neuronal, adipogenic and osteogenic lineages in vitro.

**Results:**

The NPs were characterized by transmission electron microscopy, dynamic light scattering, ultraviolet–visible spectroscopy and fluorescence spectroscopy. Covalent conjugation of the FGF2 to the IO/HSA NPs significantly stabilized this growth factor against various enzymes and inhibitors existing in serum and in tissue cultures. IO/HSA NPs conjugated to FGF2 were internalized into hBM-MSCs via endocytosis as confirmed by flow cytometry analysis and Prussian Blue staining. Conjugated FGF2 enhanced the proliferation and clonal expansion capacity of hBM-MSCs, as well as their adipogenic and osteogenic differentiation to a higher extent compared with the free growth factor. Free and conjugated FGF2 promoted the expression of neuronal marker Microtubule-Associated Protein 2 (MAP2) to a similar extent, but conjugated FGF2 was more effective than free FGF2 in promoting the expression of astrocyte marker Glial Fibrillary Acidic Protein (GFAP) in these cells.

**Conclusions:**

These results indicate that stabilization of FGF2 by conjugating the IO/HSA NPs can enhance the biological efficacy of FGF2 and its ability to promote hBM-MSC cell proliferation and trilineage differentiation. This new system may benefit future therapeutic use of hBM-MSCs.

**Electronic supplementary material:**

The online version of this article (doi:10.1186/s12951-015-0090-8) contains supplementary material, which is available to authorized users.

## Background

Magnetic nanoparticles (NPs) which are known for their very large surface area and magnetic properties (size up to 0.1 μm) have a wide range of potential applications such as drug delivery, MRI, diagnostics, hyperthermia, specific cell labeling and separation, cell tracking and bio-catalysis [1-6]. Iron oxide (IO) NPs of sizes less than approximately 50 nm are superparamagnetic (possess magnetic properties when they are exposed to external magnetic field, and lose their magnetic properties when the magnetic field is removed), allowing therefore the separation of these NPs by using high gradient magnetic columns. IO NPs are also known for their non-toxicity and biodegradability, therefore ideal for biomedical applications [7,8]. Previous studies showed that it is also possible to mark the IO NPs with a fluorescent dye, e.g., near IR (NIR) dye, which further improves the probe capabilities [9].

NIR light of 700 to 1000 nm, achieves the highest tissue penetration due to minimal absorbency of the surface tissue in this spectral region. In vivo fluorescence imaging has experienced substantial growth with the “opening” of the NIR “window” because of the development of novel NIR fluorescence probes and optical imaging instruments [10-12]. In previous studies we described the generation of IO NPs coated with Human Serum Albumin (HSA) [13]. HSA exhibits an average blood half-life of 19 days and is emerging as a versatile protein carrier for drug targeting and improving the pharmacokinetic profile of peptide or protein-based drugs [14]. These properties combined with lack of toxicity, easy availability, biodegradability and preferential uptake in tumor and inflamed tissues make the core-shell IO/HSA NPs an ideal candidate for drug targeting and delivery. In addition, another important property of the HSA coating onto the IO NPs is that its various functional groups, e.g., carboxylates, amines, hydroxyls and thiols, can easily be used through different activation methods for conjugation of the IO/HSA NPs to various biomolecules such as proteins, amino acids, antibodies, oligonucleotides, etc. [15,16]. Conjugation of proteins to IO/HSA NPs is predicted to reduce their susceptibility to chemical, enzymatic and thermal degradation, thus enhancing the protein biological efficacy [17-20]. Furthermore, it may provide a mean for sustained release of the conjugated proteins.

Bone marrow mesenchymal stem cells (BM-MSCs) are multipotent cells that can differentiate into mesenchymal and non-mesenchymal lineages. They can give rise to osteogenic, chondrogenic, adipogenic, myoegenic, hepatogenic, endothelial and neurogenic cells both *in vitro* and *in vivo* [21-26]. BM-MSCs secrete trophic factors that can promote the survival of damaged cells, as well as immunomodulatory cytokines that can suppress T-cell proliferation and function [27-31]. Because of their good proliferation, differentiation and paracrine potential, as well as their relative ease of isolation and low immunogenicity, BM-MSCs have become a main source for tissue engineering of bone, cartilage, muscle, marrow stroma, tendon, fat, and other connective tissues [32-34]. Furthermore, we and others have shown that hBM-MSC transplantation has the potential to ameliorate the symptoms of various neurodegenerative diseases, including retinal degeneration, Alzheimer's disease, Parkinson, familial amyotrophic lateral sclerosis and multiple sclerosis [29,35-37] as well as other disease such as acute liver failure [38] and pulmonary emphysema [39]. These and other successful animal studies have led to numerous clinical trials using hBM-MSC as a source for cellular therapy for treatment of heart, liver, bone and cartilage repair, foot ulcers, spinal cord injuries, peripheral nerve injuries and acute graft-versus-host disease [40-46]. Since mesenchymal stem cells comprise only 0.001-0.01% of the bone mononuclear cells, extensive *in vitro* expansion is required to obtain sufficient number of cells for clinical use [47]. Although the cells have high proliferation potential, prolonged culture expansion may reduce the cell differentiation potential. In addition, proliferation and differentiation potential varies between donors [48]. Hence enhancing cell proliferation and differentiation potential could improve their yields for clinical applications.

In addition, following transplantation of hBM-MSc there is a need to repeatedly monitor the cells in vivo in a non-invasive manner. This cannot be achieved using histological and immunohistochemical techniques that require tissue removal. We have previously shown that prelabeling of mesenchymal stem cells with IO NPs enables noninvasive *in-vivo* tracking following cell transplantation using Magnetic Resonance Imaging (MRI, [49]).

Several studies have demonstrated that supplementation of basic FGF (also known as FGF2) to BM-MSC culture medium increases cell proliferation rate and cell differentiation [50,51]. However, as the cells are cultivated at 37 degrees, rapid enzymatic degradation and protein denaturation leads to short time life of FGF2 of about 3–10 minutes and reduces its biological activity and functions [52,53]. In a previous study we showed that conjugation of FGF2 to IO/HSA NPs stabilized the factor and significantly improved its ability to promote rat nasal olfactory mucosa cell migration, growth and differentiation [54]. The present article describes a method of preparing FGF2-conjugated IO/HSA NIR fluorescent core-shell NPs that significantly stabilized the FGF2 through its covalent conjugation to the nanoparticle’s surface [55,56]. We also show that FGF2 conjugated to IO/HSA NPs is internalized by hBM-MSCs and promotes the growth and trilineage (neuronal, bone, fat) differentiation capacity of the cells at a higher extent compared with the free FGF2.

## Results and discussion

The NIR fluorescent IO/HSA NPs were prepared by nucleation followed by stepwise growth of IO thin films onto the gelatin/IO nuclei as described in the “Methods” section.

### Nanoparticles’ characterization: dry and hydrodynamic size and size distribution

**Figure 1:**
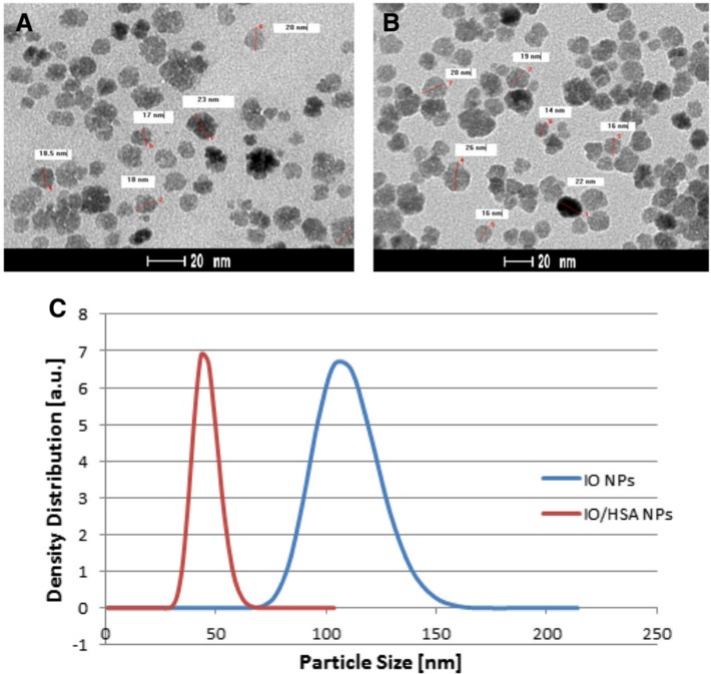
Dry and Hydrodynamic Size and Size Distribution of IO Nanoparticles. TEM images of the dry core IO **(A)** and the core-shell IO/HSA **(B)** NPs; **(C)** Hydrodynamic size and size distribution of the core IO & the core-shell IO/HSA NPs dispersed in an aqueous phase.

IO core NPs and IO/HSA core-shell NPs were both diluted with H_2_O to a concentration of 1 mg/ml and dried over a TEM grid. TEM measurements (Figure 1A,B) indicate that the size and size distribution of the core and core/shell NPs are 17 ± 1 nm and 21 ± 3 nm, respectively. Samples of IO and IO/HSA were dispersed in H_2_O and their hydrodynamic diameters were determined (using DLS) to be 103 ± 14 nm and 43 ± 5 nm, respectively (Figure 1C). These hydrodynamic measurements demonstrate that the albumin coating decreased the hydrodynamic size of the IO NPs, as clearly shown in Figure 1C. These size differences between TEM and DLS are attributed to the fact that TEM measures the dry diameter, while DLS determines the hydrodynamic diameter, which takes the hydrated layers on the particle surface into account. In addition, the difference in the hydrodynamic size of the IO core NPs and the HSA/IO core-shell NPs may indicate that the heat denatured albumin coating is more hydrophobic than the core IO NPs.

### Fluorescence spectroscopy

**Figure 2:**
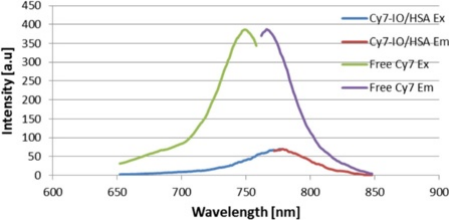
Excitation and emission of Free Cy7 and Cy7-IO/HSA NPs. The excitation and emission spectrum of Cy7-IO/HSA NPs and the free Cy7 in PBS at a final concentration of 250 ng/ml were determined using spectrofluorometer.

The excitation and emission spectrum of the Cy7-IO/HSA NPs and the free Cy7 in PBS are shown in Figure 2. The maximum fluorescence excitation of the Cy7-IO/HSA NPs and free Cy7 occurs at approximately 769 and 749 nm, respectively. The maximum fluorescence emission intensity of the Cy7-IO/HSA NPs and free Cy7 occurs at approximately 780 and 766 nm, respectively. The red-shift in the absorbance spectrum of the NIR fluorescent IO/HSA NPs compared with the free Cy7 dye is probably due to its binding to the gelatin within the IO core NPs that affects the dipole moment of the dye [20].

### Photobleaching stabilization

**Figure 3:**
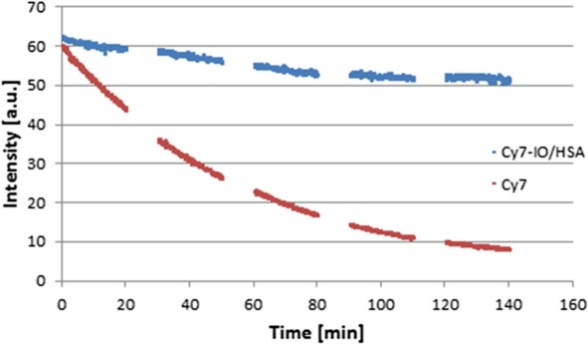
Photobleaching Stabilization of Cy7 by encapsulation. Fluorescence intensity as function of time of free Cy7 and Cy7-IO/HSA NPs following illuminated at 800 nm was measured using spectrofluorometer.

To study the fluorescence stability of the Cy7 encapsulated NPs, a photobleaching experiment was performed for free Cy7 dye and Cy7-IO/HSA, as described in the literature [14]. Both samples were illuminated at 800 nm, and their fluorescence intensities were measured, 5 cycles of 20 min each with 10 min recovery between cycles. The fluorescence intensity of the NIR fluorescent IO/HSA NPs decreased by 5% after the first cycle (t = 20 min) and by 15% after all 5 cycles (t = 140 min), while the fluorescence of free Cy7 decreased by 38% after the first cycle (t = 20 min) and by 87% after all cycles (t = 140 min), as shown in Figure 3.

The irreversible light-induced destruction of the fluorophore also known as photobleaching is affected by factors such as temperature, exposure time, oxygen, oxidizing or reducing agents and illumination levels [56]. The encapsulation of Cy7 within the NPs significantly reduced the photobleaching as demonstrated in Figure 3. Encapsulation of the dye probably protects the dye against reactive oxygen species, thereby reducing photobleaching [55,57]. Previous work in our lab showed similar results with the dye RITC conjugated to NPs [58].

### Long term stability of free versus conjugated neurotrophic factors

**Figure 4:**
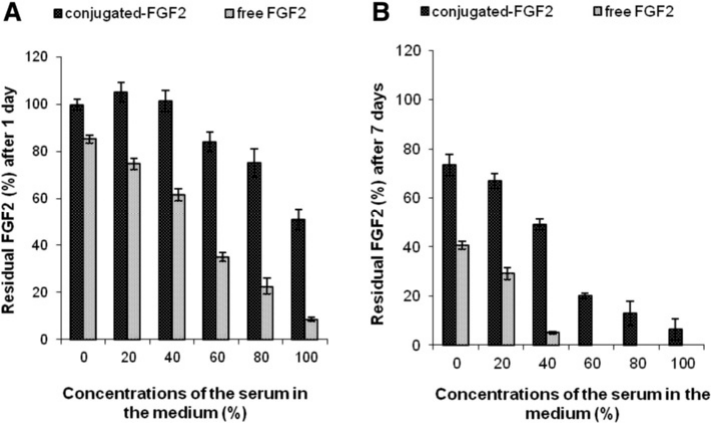
Stability of free versus conjugated-FGF2. Ten ng/ml of free or conjugated FGF2 were incubated in various concentrations of fetal calf serum (0–100 %) in the medium at 37°C according to the experimental section. The residual concentrations of the growth factors following 1 and 7 days of incubation are shown in **A** and **B**, respectively.

FGF2 was chosen to serve as a model for neurotrophic factors. The stability of the free and conjugated FGF2 against various enzymes and inhibitors existing in serum and in tissue culture was examined. The stability was tested in various concentrations of serum. Figure 4A and B indicates that the concentrations of the free and the conjugated-FGF2 decreased with time and with increasing concentration of the serum. However, the concentration of the free FGF2 decreased significantly more rapidly than that of the conjugated factor (p < 0.004). For example, the residual concentration of the conjugated factor following incubation for one day in medium containing 40, 80 and 100% serum was 101 ± 4.5, 75 ± 6.2 and 51 ± 4.2% from the initial concentrations, respectively, while the residual concentrations of the free factor were only 62 ± 2.5, 23 ± 3.5 and 9.0 ± 0.8%, respectively (Figure 4A). Examination of the residual concentrations of FGF2 remaining after incubation for one week in a medium containing 20, 40 and 60% of serum, demonstrated a dose–response relationship wherein increasing serum concentration resulted in reduced concentration of FGF2. Thus, the concentration of free factor was reduced to 29 ± 2.4, 5 ± 0.5 and 0% from the initial concentration, respectively. By contrast the concentration of the conjugated-FGF2 was significantly higher (66.9 ± 3.0, 49.1 ± 2.4 and 20.2 ± 0.9%, respectively, Figure 4B). These results indicate that the conjugated-FGF2 is significantly more stable in serum than the free factor.

### Conjugated-FGF2 promotes hBM-MSC cell expansion

**Figure 5:**
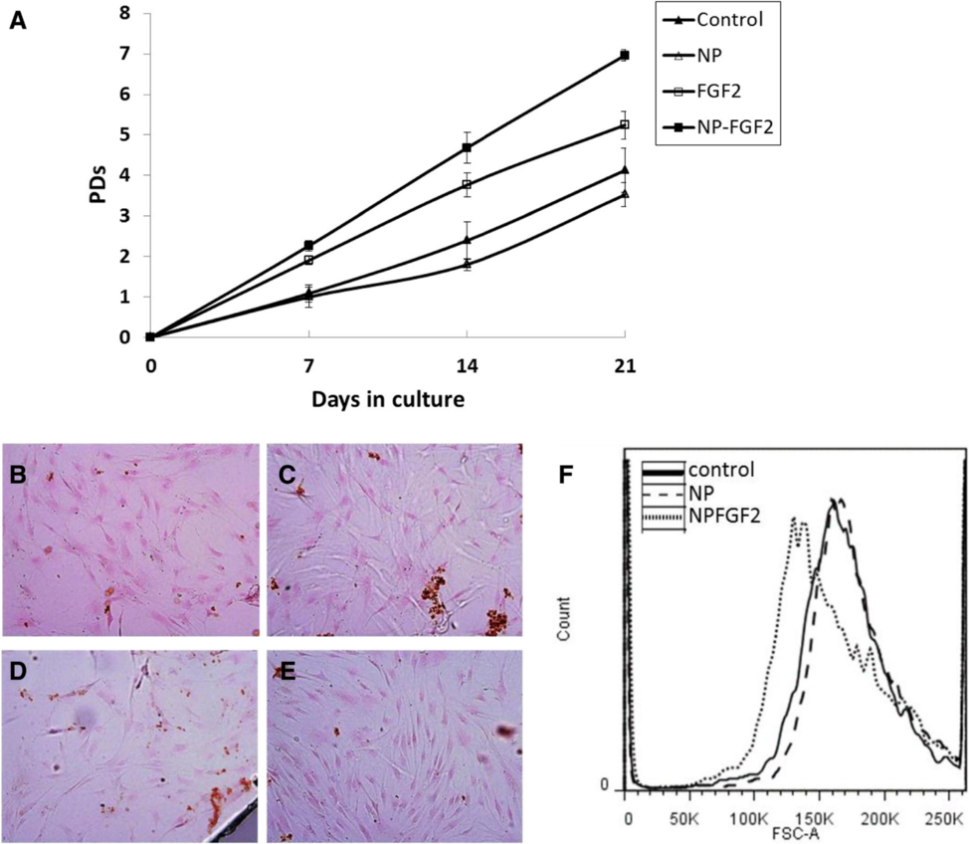
Effect of conjugated FGF2 on the growth and size of hBM-MSCs. **(A)** Cells were cultured in the absence or presence of 0.1ng/ml free FGF2, 0.1 ng/ml conjugated FGF2 or 90 ng/ml Cy7-IO/HSA NPs. Cells were passaged and counted every 7 ± 2 days. Cumulative population doublings (PDs) were calculated. **(B-E)** Light microscope images of cells grown in the absence **(B)** or presence of 0.1ng/ml free FGF2 **(C)**, 0.1 ng/ml conjugated FGF2 **(D)** or 90 ng/ml Cy7-IO/HSA NPs **(E)**, following nuclear fast red staining. Magnification- 100x. **(F)** Flow cytometry analysis of the size of hBM-MSCs treated with 50 ng/ml conjugated FGF2 or 45 μg/ml Cy7-IO/HSA for 48 hours.

To examine the effect of conjugated FGF2 on hBM-MSC expansion, the cells were subcultured for 3 passages in growth media supplemented with 0.1 ng/ml free or conjugated FGF2. As shown in previous studies [50,59], cells grown in the presence of free FGF2 demonstrated higher proliferation rates compared to control cultures (Figure 5A). Moreover, the population doubling (PD) time for hBM-MSCs expanded in the presence of conjugated FGF2 was consistently shorter than that of cells expanded in the presence of free FGF2 or control conditions at all passages (Figure 5A). Following 3 passages in the presence of conjugated FGF2, the cells reached on average 7 ± 0.14 population doublings. By contrast cells cultivated with free FGF2, non-conjugated NPs or control conditions, reached only 5.2 ± 0.3, 3.5 ± 0.3 or 4 ± 0.5 PDs, respectively (Figure 5A). The yield of cells obtained from expanding the cells in the presence of conjugated FGF2 was over 3 fold higher than that of free FGF2 and nearly 8 fold higher than the yield obtained from cells grown under control conditions (Additional file 1: Figure S1). Immunofluorescence analysis using antibody directed against the proliferating cell nuclear antigen (PCNA), demonstrated that all cells under all growth conditions were positive for this proliferation marker (data not shown). Trypan blue staining was performed in every subculturing. No dead cells were identified in any of treatments in any of passages or donors. As was previously demonstrated by others for free FGF2 [60,61], hBM-MSCs cultured in the presence of FGF2 conjugated to IO NPs were smaller compared with cells cultured under control conditions or in the presence of non-conjugated NPs (Figure 5B-F). Cell surface antigen phenotyping clearly demonstrated that hBM-MSCs cultivated for 3 passages in the presence of NP-FGF2 maintained the expression of mesenchymal cell markers CD73, CD90, CD105 and were negative for hematopoietic markers CD14, CD34 and CD45 (Additional file 2: Figure S2). Furthermore the cells maintained low levels of expression of HLA-DR, suggesting that expansion in the presences of 0.1 ng/ml conjugated FGF2 will not increase their immunogenicity in vivo. Taken together our findings suggest that expansion of hBM-MSCs in the presence of 0.1 ng/ml conjugated FGF2 substantially increases cell yields with no adverse effects on expression of mesenchymal surface markers.

### Uptake of Nanoparticles by hBM-MSCs

**Figure 6:**
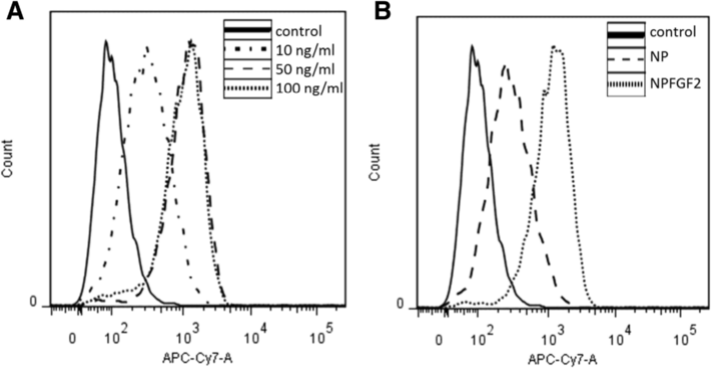
Determination of uptake of Cy7-IO/HSA NPs by hBM-MSCs using flow cytometry. Flow cytometry analysis of the uptake of Cy7-IO/HSA by hBM-MSCs non-treated (control) or treated with **(A)** FGF2 conjugated Cy7-IO/HSA in three FGF2 concentrations (10, 50 and 100 ng/ml) for 48h or with **(B)** 50 ng/ml FGF2-Cy7-IO/HSA or 45 μg/ml non-conjugated NPs.

To examine the uptake of conjugated FGF2, hBM-MSCs were incubated with increasing concentration of conjugated-FGF2 and analyzed by flow cytometry to detect the Cy7 fluorescence signal. Figure 6 demonstrates efficient uptake of the conjugated FGF2 by the cells following 48 h incubation with Cy7-IO/HSA-FGF2 NPs. Quantification of Cy7 positive cells revealed that 35.6% of the cells incubated with 10 ng/ml conjugated FGF2 (9 μg/ml Cy7-IO/HSA NPs) were Cy7 positive (Figure 6A). In cells cultured in the presence of 50 ng/ml conjugated FGF2 (45 μg/ml Cy7-IO/HSA NPs), 97% of the cells were Cy7-positive. Similar results were obtained in cells cultured with 100 ng/ml conjugated FGF2 (90 μg/ml Cy7-IO/HSA NPs), with 96% of the cells positive for Cy7 (Figure 6A). Only 26% or 28.4% of the cells were positive for Cy7 following incubation with 9 or 45 μg/ml free Cy7-IO/HSA NPs, respectively (Figure 6B and data not shown). However, when cells were cultured with 90 μg/ml Cy7-IO/HSA NPs, 90% of the cells were Cy7 positive, suggesting that at low concentrations the Cy7-IO/HSA internalization was mediated at least in part by endocytosis of FGF2, but at high concentrations the cells can internalize the NPs in a non-FGF2 dependent pathway.

**Figure 7:**
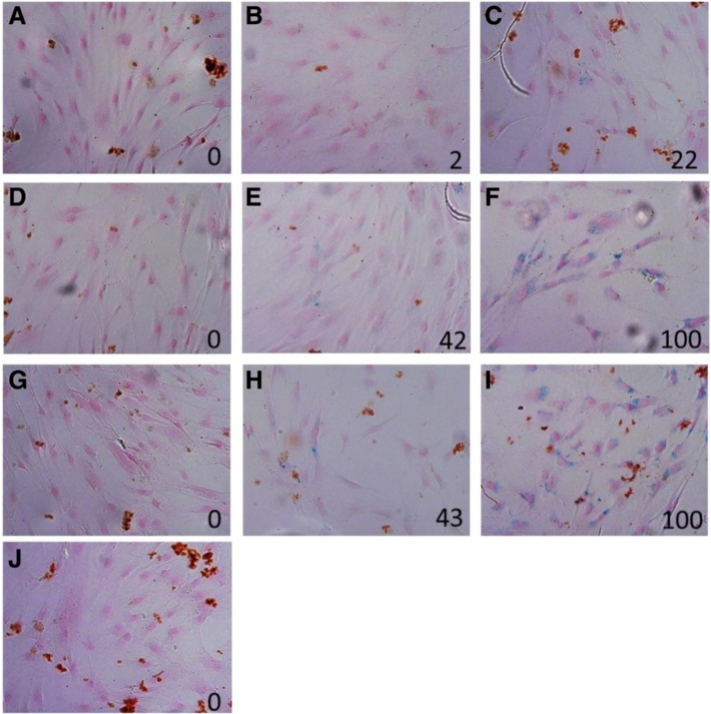
Determination of uptake of Cy7-IO/HSA NPs by hBM-MSCs using Prussian Blue iron staining. Cells were grown in the absence (control, **J**) or presence of 10 ng/ml **(A,C)**, 50 ng/ml **(D,F)**or 100 ng/ml **(G,I)** free FGF2 **(A, D, G)** or conjugated-FGF2 NPs **(C, F, I)** , or non-conjugated IO/HSA NPs at concentration of 9 μg/ml **(B)**, 45 μg/ml **(E)** or 90 μg/ml **(H)**. Following fixation in 4% PFA, cells were stained with Prussian Blue iron stain (blue) and counter stained with Nuclear Fast Red (red). The percentage of Prussian Blue positive cells was calculated from 3 microscopic fields and is indicated at the bottom of each picture. Magnification (×200).

The cellular uptake of these nanoparticles into the hBM-MSCs was also confirmed by Prussian Blue iron staining as shown in Figure 7. This figure demonstrates blue granules inside the cells incubated with 10–100 ng/ml FGF2 conjugated to Cy7-IO/HSA NPs for 48 h, indicating the accumulation of conjugated NPs in the cells. Increasing the concentration of conjugated FGF2 enhanced the amount of positively stained cells. Thus, at 10 ng/ml conjugated FGF2, nearly 23% of the cells were positively stained with Prussian Blue (Figure 7C). By contrast, 100% of cells incubated with 50 or 100 ng/ml conjugated FGF2 were positively stained with this dye (Figure 7F,I).

Cells incubated with non-conjugated IO/HSA NPs were also positive for Prussian Blue staining, but to a lower extent compared with the FGF2-conjugeted IO/HSA NPs (Figure 7B,E,H). This data strongly suggest that at concentration of 50 ng/ml and lower, the majority of FGF2-conjuagted NPs were most probably internalized by the cells via receptor-mediated endocytosis. Endocytosis of FGF2-conjugated NPs could be mediated by FGF receptor 1 (FGFR1) that is expressed in hBM-MSCs and mediates FGF2 internalization and signaling [62,63]. Our findings are supported by various studies that demonstrated that conjugating a variety of ligands to NP surfaces facilitates receptor-mediated endocytosis of the NPs [64]. TUNEL staining revealed that there were no apoptotic cells in cultures supplemented with 100 ng/ml conjugated or free FGF2, 90 μg/ml non-conjugated NPs or control cells, supporting the biocompatibility of the IO/HSA NPs (data not shown).

### Effect of conjugated FGF2 on the clonal expansion capacity of hBM-MSCs

**Figure 8:**
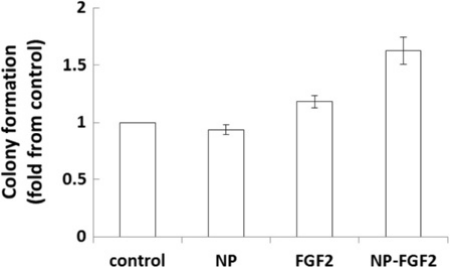
Enhanced clonal expansion capacity of hBM-MSCs in the presence of conjugated FGF2. Human BM-MSCs were seeded in 6 well plates and incubated in growth media alone (control) or growth media supplemented with 50 ng/ml free or conjugated FGF2, or free NPs. Media was change every 3 days. Seven days post seeding, colonies were counted following extensive washing and Giemsa staining. Data is presented as fold increase in colony number compared with control (mean ± SE of 3 experiments in duplicates or triplicates using cells from 3 different donors).

One of the characteristic features of hBM-MSCs is generation of colonies when plated at low densities and the efficacy of colony formation is indicative of the cell proliferation potential [65]. We compared the effect of supplementing the growth media with free or conjugated FGF2 on the clonal expansion capacity of the cells. Non-conjugated NPs were added as control. Addition of 45 μg/ml free IO/HSA NPs to the growth media had no significant effect on cloning efficiency (Figure 8 and Additional file 3: Figure S3), further demonstrating the biocompatibility of these nanoparticles. Supplementation of conjugated FGF2 significantly enhanced colony formation by the hBM-MSC by nearly 2 fold compared with free IO/HSA NPs and by 1.5 fold compared with free FGF2. The difference between the 3 supplements was statistically significant (p < 0.001). These data suggest that conjugated FGF2 is more effective in enhancing cloning efficiency of hBM-MSC than free FGF2.

### Effect of conjugated FGF2 on neurogenic differentiation

**Figure 9:**
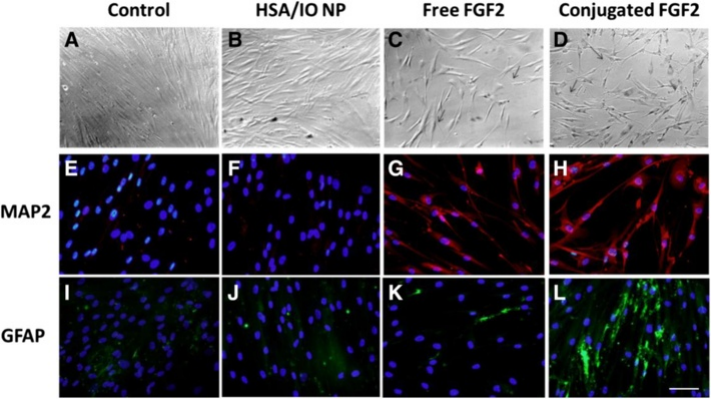
Conjugated FGF2 enhances neurogenic differentiation of hBM-MSCs. Human BM-MSCs were seeded on cover slips and incubated in neurogenic growth media (control, **A,E,I**) or neurogenic growth media supplemented with HSA/IO NPs **(B,F,J)**, 50 ng/ml free FGF2 **(C,G,K)** or 50 ng/ml conjugated FGF2 **(D,H,L)**. Media were changed every 3 days. Fourteen days post seeding, cells were fixed, photographed (top row) or stained with antibodies directed against MAP2 (red, middle row) or GFAP (green, bottom row), and nuclei were counterstained with DAPI (blue). Scale bar 100μm. Arrows in panels **C** and **D** point to elongated processes observed only in cells cultured with free or conjugated FGF2.

Human BM-MSCs are multipotent cells that can differentiate into a variety of cell types, including neuronal, bone and fat cells. We first examined the effect of conjugated FGF2 on neurogenic capacity of hBM-MSCs by monitoring cell morphology as well as the expression of two neuronal differentiation markers - Microtubule-Associated Protein 2 (MAP2), a marker of neuronal differentiation that is expressed exclusively during the neuronal differentiation of neural precursor cells, and the astrocyte marker Glial Fibrillary Acidic Protein (GFAP). As shown in Figure 9, cells incubated for 14 days in neuronal differentiation media with no supplementation of FGF2 (control) or in the presence of free IO/HSA NPs failed to present the morphological changes characteristic of neuronal cells or to express the MAP2 and GFAP markers (Figure 9 A,B,E,F,I,J). By contrast, cells supplemented with either free FGF2 or conjugated growth factor at 50ng/ml presented morphologic changes typical to neuronal cells, with bipolar morphology and elongated processes (arrows in bright field images point to elongated processes, Figure 9C,D). Furthermore, 100% of cells incubated for 2 weeks with either free or conjugated FGF2 expressed MAP2 (Figure 9G,H). Conjugated FGF2 was over 3 fold more efficient in inducing the expression of the astrocyte marker GFAP in these cells compared with the free FGF2 (Figure 9K,L). Thus, 11.4% of cells incubated with free FGF2 were positive for GFAP staining whereas 35% of the cells incubated with conjugated FGF2 expressed GFAP. Taken together our data suggest that conjugated FGF2 enhances hBM-MSC neurogenic potential and was more efficient than free FGF2 in promoting differentiation to glial cells.

### Effect of conjugated FGF2 on adipogenic differentiation

**Figure 10:**
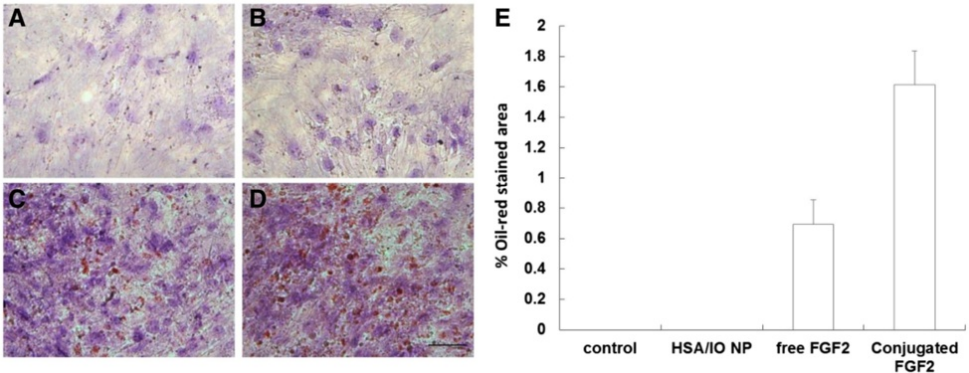
Effect of free and conjugated FGF2 on adipogenic differentiation of hBM-MSCs. Cells were grown in adipogenic differentiation media (**A**, control) supplemented with 3 ng/ml free FGF2 **(C)** or conjugated FGF2 **(D)** or free IO/HSA NPs **(B)**, for 14 days. Cells were stained with Oil-red and nuclei were counterstained with hematoxylin. Bar-200μm. **(E)** To evaluate adipogenic differentiation, the percentage of Oil-red positive area/total area × 100 was calculated in 3 random non overlapping fields of triplicates for each supplement.

To test the effect of conjugated FGF2 on the adipogenic differentiation potential of hBM-MSCs, cells were grown for 14 days in adipogenic media with or without supplementation of 3 ng/ml free FGF2 or conjugated FGF2, or free IO/HSA NPs. To evaluate adipogenesis, cultures were stained with Oil red that stains lipid droplets in adipocytes. As shown in Figure 10, conjugated FGF2 was over 2 fold more efficient in promoting adipogenic differentiation compared with free FGF2 (p < 0.0001).

### Effect of conjugated FGF2 on Osteogenic differentiation

**Figure 11:**
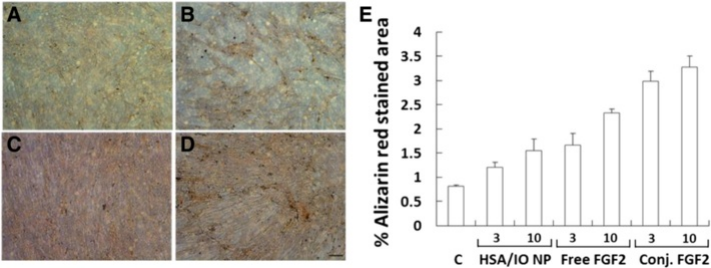
Effect of free and conjugated FGF2 on osteogenic differentiation of hBM-MSCs. Cells were grown in osteogenic differentiation media (**A**, control) supplemented with 10 ng/ml free FGF2 **(C)** or conjugated FGF2 **(D)** or free IO/HSA NPs **(B)**, for 14 days. Cells were stained with Alizarin-red. Bar-200μm. **(E)** To evaluate the osteogenic differentiation capacity of each supplement, the Alizarin-red O positive area/total area x100 was calculated in 3 areas from triplicate samples for control (c) or each supplement at 3 or 10 ng/ml as indicated in the graph.

The effect of conjugated FGF2 on osteogenic differentiation was examined by treating the cells for 14 days in osteogenic induction media supplemented with 3 ng/ml or 10 ng/ml free FGF2 or conjugated FGF2 or free IO/HSA NPs, as control. Following fixation, cells were stained with Alizarin Red S solution to visually detect the presence of mineralization. Conjugated FGF2 at 3 ng/ml was 2 fold more efficient in promoting osteogenic differentiation compared with free FGF2 at this concentration, and slightly more efficient than 10 ng/ml free FGF2 (Figure 11). Statistical analysis suggested a highly significant difference between the free and conjugated FGF2 treatments (p < 0.001) and between the different treatment concentrations (p = 0.025). Together, the effects of concentration and treatment explained a high proportion of the observed osteogenic differentiation (R squared = 0.864). There was no interaction between the concentration and treatment type parameters.

## Conclusions

Taken together we have shown here that IO/HSA NPs are biocompatible and that FGF2-conjugated IO/HSA NPs significantly enhanced hBM-MSC growth and trilineage differentiation compared with the same concentration of free FGF2. Our findings suggest that these FGF-coupled NPs may possibly be used for expanding hBM-MSCs and enhancing their differentiation potential for future therapeutic use. As the cells endocytose the FGF2-IO/HSA NPs, it is very likely that these NPs will facilitate in-vivo detection of transplanted cells using MRI and as an added benefit, the labeled cell may be imaged by NIR fluorescence using optical coherence tomography (OCT) and other in vivo imaging systems equipped with NIR fluorescence. In this work we tested the biological activity of conjugated FGF2 as there is a vast amount of literature supporting the use of this growth factor for enhancing growth and differentiation of hBM-MSCs. The effect of supplementing the growth media of hBM-MSCs with FGF2-conjugated IO/HSA NPs on the cell therapeutic effect in vivo in animal models of neuroretinal degeneration will be investigated in future studies.

In future work we will also test the effect of other conjugated factors such as Bone Morphogenetic Protein 1 (BMP1) for bone defect applications and Heparin binding Epidermal Growth Factor-like Growth Factor (HB-EGF) for neuroretinal degeneration applications.

## Methods

### Materials

The following analytical-grade chemicals were purchased from commercial sources and used without further purification: bicarbonate buffer (BB; 0.1M, pH 8.4), ferric chloride hexahydrate, hydrochloric acid (1 M), sodium hydroxide (1 M), sodium nitrate, Triton X-100, gelatin from porcine skin, human serum albumin (HSA), NHS-Cy7, rhodamine isothiocyanate (RITC), divinyl sulfone (DVS), triethylamine (TEA), D-glucose from Sigma (Israel); FGF2 ELISA kit and recombinant human FGF2 from PeproTech (Israel); Midi-MACS magnetic columns from Almog Diagnostic (Israel); phosphate-buffered saline (PBS free of Ca+2 and Mg+2; 0.1 M, pH 7.4) from Biological-Industries (Israel); tissue culture plates (96 wells) and plastic tips from Greiner bio-one (Germany); Water was purified by passing deionized water through an Elgastat Spectrum reverse osmosis system (Elga, High Wycombe, UK). All tissue culture reagents were from Biological Industries (Israel). B27 and DAPI were from Invitrogen. Dexamethasone, insulin, β-glycophostphate, ascorbate phosphate, neuron-specific microtubule-associated protein 2 mouse monoclonal antibody and dyes were from Sigma. Glial Fibrillary Acidic Protein rabbit monoclonal antibody was from Cell Signaling. TUNEL TMR Red was from Roche. Secondary antibodies were from Jackson ImmunoResearch. PCNA (pc1-0) mouse monoclonal IgG2a was from Santa Cruz, USA.

### Preparation of the non-fluorescent and fluorescent core IO nanoparticles

Core IO NPs of narrow size distribution were prepared by nucleation in the initial part, followed by stepwise controlled growth of IO thin films onto gelatin/IO nuclei. Briefly, IO NPs of 18 ± 1 nm diameter were prepared by adding FeCl_2_ solution (10 mmol/5 ml H_2_O, 1 N HCl 0.5ml) to 80 ml aqueous solution containing 240 mg gelatin (during the whole procedure, the aqueous suspension is agitated at 60°C), followed by NaNO_3_ solution (7 mmol/5 ml H_2_O). Next, 1N NaOH aqueous solution was added up to pH 9.5. This procedure was repeated three times with 10 min intervals. The formed magnetic NPs were then washed from excess reagents with water using high gradient magnetic field (HGMF) technique. As soon as the washing step was completed, the column was removed from the magnetic field and the NPs were eluted by adding an aqueous bicarbonate buffer (BB, 0.1M, pH = 8.3) [66]. NIR core IO NPs were prepared similarly, by substituting the gelatin for gelatin covalently conjugated with NHS Cy7 to obtain NIR-IO NPs [20].

### HSA coating onto the fluorescent IO core nanoparticles

HSA coating was performed by shaking the aqueous suspension of the fluorescent IO NPs with 10% HSA (MW ~66,000) at 75°C for 12h. The HSA coated NPs were then washed from excess reagents by magnetic columns with PBS (pH = 7.4).

### Activation of the fluorescent IO/HSA nanoparticles

Activation of the fluorescent IO/HSA NPs was performed by functionalization of these NPs with excess DVS. One double bond created a covalent bond with the amino groups of the HSA coating onto the fluorescent IO NPs. The residual activated double bond was then used for covalent binding of ligands containing primary amino groups. Briefly, 20 μl of DVS were added to 1 ml of the fluorescent IO/HSA NPs (5 mg/ml) dispersed in the BB continuous phase. The dispersion was then shaken for 12h at 60°C and the remaining free DVS was then washed from the obtained DVS-conjugated NPs using magnetic columns with BB.

### Conjugation of FGF2 to the activated fluorescent IO/HSA nanoparticles

Bioactive ligands such as amino acids, proteins, antibodies and more can be easily conjugated to the DVS activated NPs. Briefly, 200 μl of dissolved FGF2 (0.1 mg/ml) were mixed with 200 μl of the DVS activated fluorescent IO/HSA NPs (5 mg/ml) dispersed in BB (0.1M, pH = 8.3). Next, the dispersion was shaken at room temperature for 60 min in order to allow the nucleophilic attack of primary amino groups (from the bioactive ligand) on the DVS-IO/HSA NPs. Blocking of residual activated DVS groups was then performed with glycine, by adding glycine (1% w/v) and mixing the dispersion for additional 30 min at room temp. Excess of unbound ligands were then removed by magnetic columns and the FGF2 conjugated IO/HSA NPs were then eluted with PBS (pH = 7.4).

### Transmission Electron Microscopy (TEM)

The TEM image provides direct information on the dry particle shape and size, in which approximately 200 NPs were measured to determine its average size. The core IO NPs and the core-shell IO/HSA NPs were diluted with H_2_O to a concentration of 1 mg/ml, dripped on a TEM grid and then dried.

### Dynamic Light Scattering (DLS)

Dynamic light scattering measures Brownian motion and relates the intensity fluctuations in the scattered light to the size and size distribution of the particles in its hydrated shape. The fluorescent core IO NPs and core-shell IO/HSA NPs were diluted with H_2_O inside a cuvette and the average diameter was then measured, while each measurement was repeated 5 times.

### Spectrofluorometer

Spectrofluorometer uses the fluorescent properties of a molecule to provide information about their concentration and fluorescence intensity, both excitation and emission, in different wavelength. Cy7 and Cy7-IO NPs were diluted with PBS to a concentration of 250 ng/ml of the dye, followed by emission, excitation and stability measurements.

### Enzyme-Linked ImmunoSorbent Assay (ELISA)

Enzyme-linked immunosorbent assay (ELISA) is commonly used to determine if a particular protein is present in a sample and its concentration [67]. In the present work the concentration of the free and conjugated FGF2 was determined by FGF2 ELISA kit (PeproTech, Israel) based on a calibration curve of known concentrations of free FGF2, according to the literature and following manufacturer’s instructions [67]. Samples of the Cy7-IO/HSA-DVS-FGF NPs were diluted with ELISA assay diluent to 3 different NPs' concentrations, each concentration was tested in triplicates and the mean value was calculated. The concentration of the bound FGF2 was determined from a calibration curve of free FGF2 and found to be 1.1 μg/mg core-shell NPs.

### Comparative stability studies of free versus conjugated FGF2

For stability measurements, free or conjugated-FGF2 were incubated (10 ng/ml) in various concentrations of fetal calf serum in the medium (0–100% of non-heated serum diluted in medium) at 37°C for 1 and 7 days. The concentration of the residual free and conjugated-FGF2 was then determined by FGF2 ELISA kit.

### Production of BM-MSCs

Fresh human Bone Marrows Mesenchymal Stromal Cells (hBM-MSCs) were collected from 6 healthy donors in the operating room at The Sheba Medical Center, Tel-Hashomer, under sterile conditions. The research was approved by the institutional review board at the Sheba Medical Center. Bone marrow mononuclear cells were separated by Ficoll gradient (1.077g/dl) according to the manufacturer instructions and were seeded in tissue culture flasks with culture media containing low-glucose Dulbecco’s Modified Eagle’s Medium (DMEM) supplemented with 15% FCS, 100U/ml penicillin, 100 ug/ml streptomycin and 2mM L-Glutamine. Tissue culture media was changed after 48 h and then twice a week until 70-80% confluence was reached. Trypan blue staining was performed in every subculturing.

### Expansion of hBM-MSCs

Cell expansion in the presence of different supplements was tested in cells derived from 3 BM donors. Cells were grown for 3 passages between passages 2–5. Cells, at concentration of 5 × 10^3^ cell/well, were seeded in 6 well plates in the presence of 0.1 ng/ml free FGF2 or conjugated FGF2 or 90 ng/ml Cy7-IO/HSA NPs in duplicates. Cells were subcultured every 7 ± 2 days and reseeded at 5 × 10^3^ cells per well. Since all plates for each donor were subcultured at the same time for each passage, control and non-conjugated NPs cultures were less confluent than their FGF-treated counterparts during subculturing. To evaluate cell morphology and size, cells were seeded on cover slips and stained with Nuclear Fast Red or analyzed by flow cytometry.

### Flow cytometry analysis

Cell surface antigen phenotyping was performed by flow cytometry (FACSCalibur, Becton-Dickinson) using antibodies directed against CD14, CD34, CD45, CD73, CD90, CD105 and HLA-DR to confirm mesenchymal cell phenotype [29,35-37].

The uptake of Cy7 within cells was evaluated by FACSAria III (BD) cell sorting. In order to maximize cell viability and minimize mechanical perturbations, we set the flow rate to 1.1 (minimum). For Cy7 analysis 633nm excitation laser was used with a filter. Data were processed by FlowJo v7.6.4.

### Cellular uptake of NPs

Cells were seeded on coverslips precoated with MSC- attachment solution following manufacturer instructions (Biological Industries, Israel). After 24h, NPs were added to cell growth media for 48hr followed by fixation with 4% paraformaldehyde (PFA). Cells were stained with Prussian Blue iron stain and Nuclear Fast Red and visualized by light microscopy (Olympus BX51).

### Colony formation assay

For Colony Forming Unit-Fibroblasts (CFU-F) assay, cells were seeded in 6 well plates (250 cell/well) in growth medium. Medium was changed every 3 days. Colonies were formed, analyzed and counted within 7 days after seeding. Cells were washed to remove non adherent colonies. Colonies were fixed in methanol, stained with Giemsa stain, and manually counted. All counting were done in a masked fashion.

### Adipogenic differentiation assay

For induction of adipogenic differentiation, cells were cultured for two weeks in growth medium supplemented with 0.6M dexamethasone and 10 mg/l Insulin. Oil-Red-O staining was performed to identify the adipogenic cells followed by hematoxylin counter staining.

### Osteogenic differentiation assay

For induction of osteogenic differentiation, cells were cultured for two weeks in growth medium supplemented with 0.1M dexamethasone, 10mM β-glycophostphate and 50 ng/ml ascorbate phosphate. Alizarin red staining was performed to identify the osteogenic cells.

### Neurogenic differentiation assay

To induce neurogenic differentiation, cells were grown in DMEM supplemented with 0.5% B27, 1% fetal bovine serum, 5% horse serum, 0.5mM retinoic acid, 20 ng/ml epidermal growth factor and 50 ng/ml nerve growth factor. After two weeks, cells were fixed in 4% PFA, and immunostained with neuron-specific Microtubule-Associated Protein 2 (MAP2) mouse monoclonal antibody or Glial Fibrillary Acidic Protein (GFAP) rabbit monoclonal antibody.

### Statistical analysis

A general linear model was used using the donor, replicate and treatment type as independent parameters and differentiation score or colony number as the dependent variables. In the analysis of adipose differentiation the score was normalized after logarithmic transformation. Equality of variance was tested and maintained in all analyzes. We used the Bonferroni correction for post hoc analyses.

## Additional files

Additional file 1: Figure S1. Growth curves for hBM-MSCs grown with conjugated FGF2. Cells were cultured in the presence of absence of 0.1 ng/ml free FGF2, 0.1 ng/ml conjugated FGF2 or 90 ng/ml Cy7-IO/HSA NPs. Cells were passaged and counted every 7±2 days and number of live cells was calculated following Trypan blue staining. Additional file 2: Figure S2. Cell-Surface markers of hBM-MSCs following expansion in the presence of conjugated FGF2. Human BM-MSCs were expanded in the absence or presence of 0.1 ng/ml conjugated FGF2 for 3 passages. Flow cytometry analysis was performed using antibodies directed against CD14, CD34, CD45, CD73, CD90, CD105 and HLA-DR. Non labeled cells (non shaded), Isotype-matched IgG controls (dash-lined) and labeled cells (shaded) curves are shown. A minimum of 10,000 events was recorded. Additional file 3: Figure S3. Clonal expansion capacity of hBM-MSCs in the presence of conjugated FGF2. Human BM-MSCs from 3 donors were seeded in duplicates or triplicates in 6 well plates and incubated in growth media alone (control) or growth media supplemented with free IO/HSA NPs, free or conjugated FGF2 at concentration of 50 ng/ml. Media was change every 3 days. Seven days post seeding, colonies were counted following extensive washing and Giemsa staining. Data is presented as colony number per 100 cells (mean ± SE). For donor B colony expansion with non conjugated NPs was not determined.
