# Supplementary figures and images for: Bioactive magnetic near Infra-Red fluorescent core-shell iron oxide/human serum albumin nanoparticles for controlled release of growth factors for augmentation of human mesenchymal stem cell growth and differentiation
Source: J Nanobiotechnology. 2015 May 7;13:34. doi: 10.1186/s12951-015-0090-8 (PMC4432958; doi:10.1186/s12951-015-0090-8)

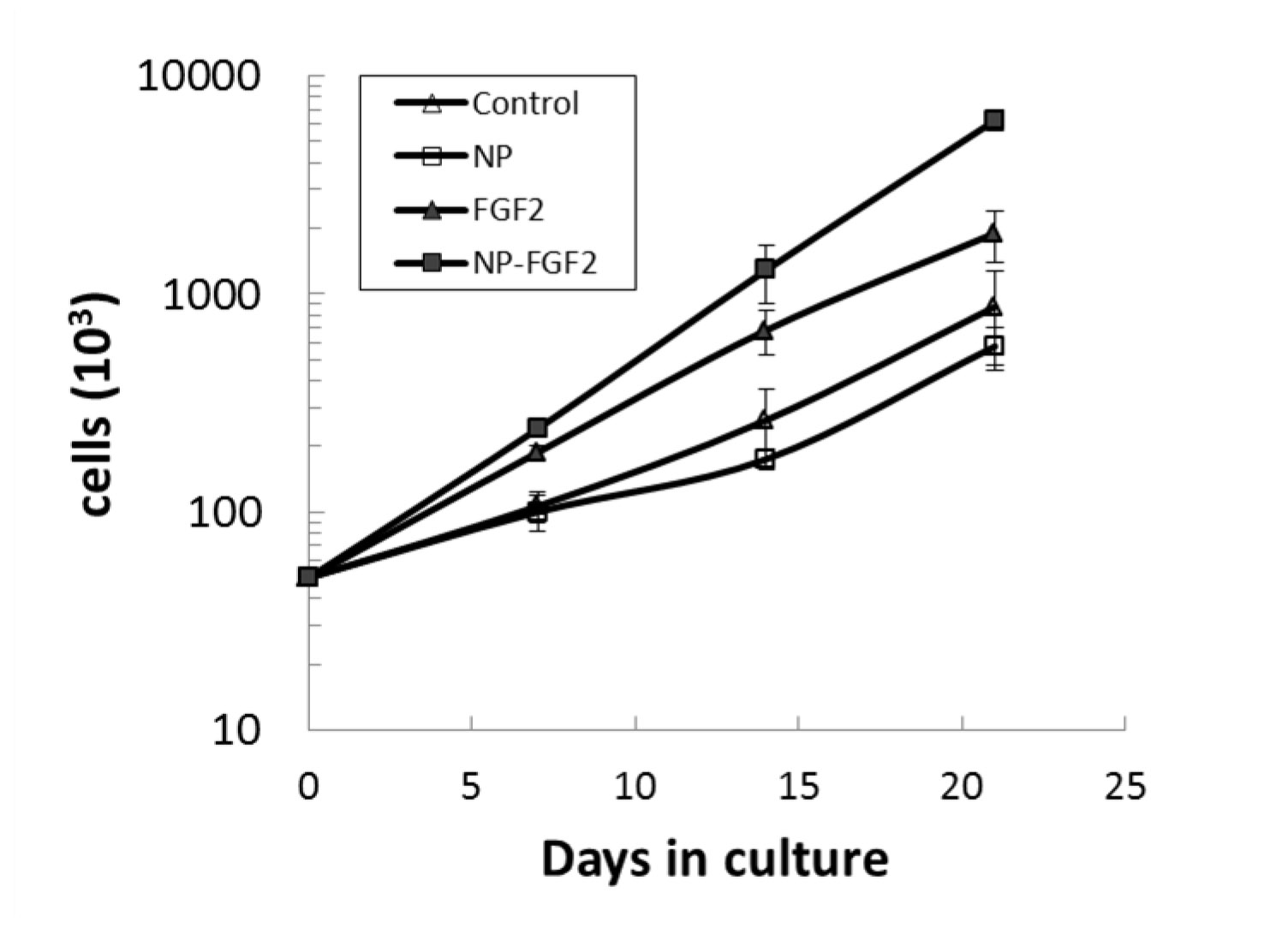

Supplement: Additional file 1: Figure S1. — Growth curves for hBM-MSCs grown with conjugated FGF2. Cells were cultured in the presence of absence of 0.1 ng/ml free FGF2, 0.1 ng/ml conjugated FGF2 or 90 ng/ml Cy7-IO/HSA NPs. Cells were passaged and counted every 7±2 days and number of live cells was calculated following Trypan blue staining. [file 12951_2015_90_MOESM1_ESM.jpeg]

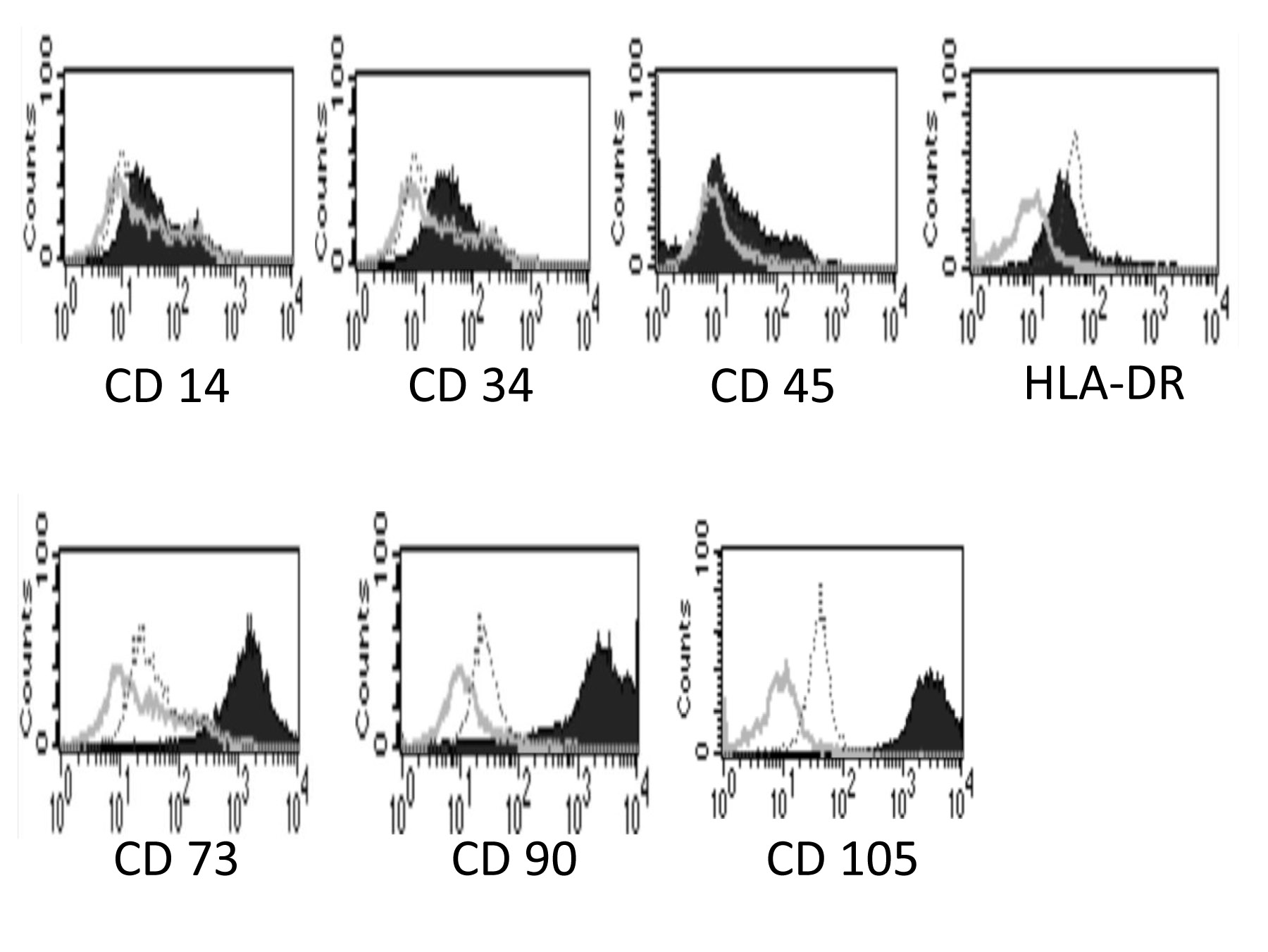

Supplement: Additional file 2: Figure S2. — Cell-Surface markers of hBM-MSCs following expansion in the presence of conjugated FGF2. Human BM-MSCs were expanded in the absence or presence of 0.1 ng/ml conjugated FGF2 for 3 passages. Flow cytometry analysis was performed using antibodies directed against CD14, CD34, CD45, CD73, CD90, CD105 and HLA-DR. Non labeled cells (non shaded), Isotype-matched IgG controls (dash-lined) and labeled cells (shaded) curves are shown. A minimum of 10,000 events was recorded. [file 12951_2015_90_MOESM2_ESM.jpeg]

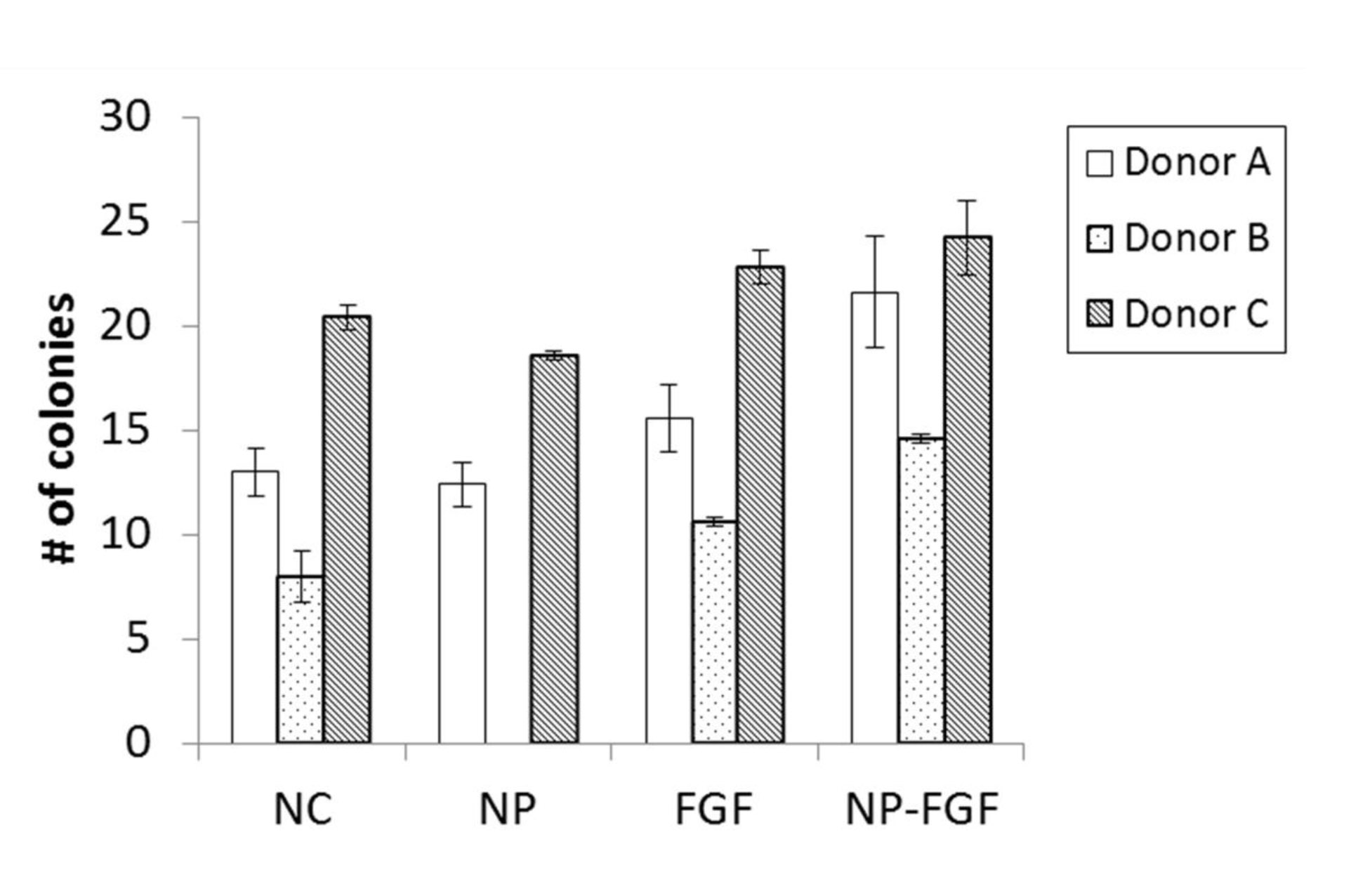

Supplement: Additional file 3: Figure S3. — Clonal expansion capacity of hBM-MSCs in the presence of conjugated FGF2. Human BM-MSCs from 3 donors were seeded in duplicates or triplicates in 6 well plates and incubated in growth media alone (control) or growth media supplemented with free IO/HSA NPs, free or conjugated FGF2 at concentration of 50 ng/ml. Media was change every 3 days. Seven days post seeding, colonies were counted following extensive washing and Giemsa staining. Data is presented as colony number per 100 cells (mean ± SE). For donor B colony expansion with non conjugated NPs was not determined. [file 12951_2015_90_MOESM3_ESM.jpeg]
